# CEP-1347 Enhances the Sensitivity of Ovarian Clear Cell Carcinoma Cells to Navitoclax by Downregulating MCL1

**DOI:** 10.3390/ijms27209261

**Published:** 2026-10-19

**Authors:** Yasufumi Ito, Yurika Nakagawa-Saito, Kazuki Nakamura, Shuhei Suzuki, Yuta Mitobe, Senri Takenouchi, Keita Togashi, Asuka Sugai, Manabu Seino, Tsuyoshi Ohta, Satoru Nagase, Chifumi Kitanaka, Masashi Okada

**Affiliations:** 1 Department of Molecular Cancer Science, Yamagata University School of Medicine, 2-2-2 Iida-nishi, Yamagata 990-9585, Japan; 2 Department of Obstetrics and Gynecology, Yamagata University School of Medicine, 2-2-2 Iida-nishi, Yamagata 990-9585, Japan; 3 Department of Neurosurgery, Yamagata University School of Medicine, 2-2-2 Iida-nishi, Yamagata 990-9585, Japan; 4 Department of Clinical Oncology, Yamagata Prefectural Shinjo Hospital, 720-1 Kanazawa, Shinjo 996-8585, Japan; 5 Department of Ophthalmology and Visual Sciences, Yamagata University School of Medicine, 2-2-2 Iida-nishi, Yamagata 990-9585, Japan

**Keywords:** epithelial ovarian cancer, myeloid cell leukemia 1, drug repositioning, drug repurposing, KT7515

## Abstract

Ovarian clear cell carcinoma (OCCC) is a rare, but highly malignant subtype of epithelial ovarian cancer that is characterized by inherent chemoresistance and limited treatment options, underscoring the need for effective and novel therapeutic approaches. We previously reported the cytotoxic effects of the combination of CEP-1347, a kinase inhibitor that induces cytostatic effects by activating the p53-p21 pathway, and navitoclax in OCCC cells; however, the mechanisms underlying this sensitization remain unclear. We herein demonstrated that the CEP-1347/navitoclax combination induced cell death in OCCC cells with different *TP53* gene statuses. Instead, a Western blot analysis of anti-apoptotic factors suggested that CEP-1347 sensitized OCCC cells to navitoclax by inhibiting MCL1 expression. In support of this, the downregulation of MCL1 sensitized OCCC cells to navitoclax, whereas MCL1 overexpression attenuated CEP-1347/navitoclax-induced cell death. Unlike S63845, an MCL1-targeting BH3 mimetic that sensitized normal cells as well as OCCC cells, CEP-1347 preferentially sensitized OCCC cells to navitoclax without affecting normal cells. These results suggest the potential of CEP-1347-based combination treatment, which preferentially sensitizes OCCC cells to navitoclax by downregulating MCL1, as a safer alternative to MCL1-targeted BH3 mimetics, and provide support for further investigations on this combination as a therapeutic strategy for OCCC.

## 1. Introduction

Ovarian cancer is one of the most lethal gynecologic malignancies and the eighth most common cancer among women worldwide. Despite treatment, the 5-year overall survival rate for advanced-stage disease remains at only 10–40% [1]. Epithelial ovarian cancer (EOC), which accounts for the majority of ovarian cancers, comprises several histological subtypes, each exhibiting distinct molecular characteristics and responses to therapy [1]. High-grade serous ovarian cancer (HGSOC) is the predominant subtype, accounting for approximately 70% of EOC cases, whereas ovarian clear cell carcinoma (OCCC) is a rare subtype, accounting for only 10% of cases globally; however, OCCC accounts for approximately 25% of EOC cases in East Asia, including Japan, and exhibits biological characteristics that are distinct from those of HGSOC [2,3]. OCCC is less sensitive to conventional chemotherapy, including platinum-based regimens, than HGSOC, and its prognosis is poor in the advanced stage. Since treatment decisions for OCCC are often extrapolated from clinical trial data dominated by HGSOC, a large unmet medical need persists, highlighting the importance of developing novel therapeutic approaches that specifically target OCCC [2,4].

We previously reported that CEP-1347, a small-molecule kinase inhibitor originally developed for the treatment of Parkinson’s disease and for which human safety and pharmacokinetic information have already been obtained through phase 2/3 clinical trials [5,6,7], potently inhibited the proliferation of cancer cells derived from various cancer types, including OCCC, at clinically relevant concentrations in vitro, without affecting normal cells [8,9,10]. Furthermore, CEP-1347 exhibited antitumor activity against xenograft tumors in vivo without compromising host health [11]. The anti-proliferative effects of CEP-1347 as a single treatment are primarily mediated by a cytostatic mechanism involving the activation of the p53–p21 pathway through the inhibition of MDM4 expression, with a limited contribution from cytotoxic mechanisms [8,9,10,11]. However, we previously demonstrated that CEP-1347 induced cellular senescence and strongly promoted cell death when combined with the senolytic drug, navitoclax (ABT-263) [12], in OCCC and glioma stem cells [13,14]. While the cytostatic effects of CEP-1347 involve the MDM4–p53–p21 axis, the mechanisms by which CEP-1347 promotes navitoclax-induced cell death remain unclear. Therefore, we herein investigated the molecular mechanisms underlying CEP-1347-induced enhancements in the sensitivity of OCCC cells to navitoclax.

## 2. Results

### 2.1. The Combination of CEP-1347 and Navitoclax Induces Apoptotic Cell Death Preferentially in OCCC Cells with Different TP53 Gene Statuses

**Figure 1:**
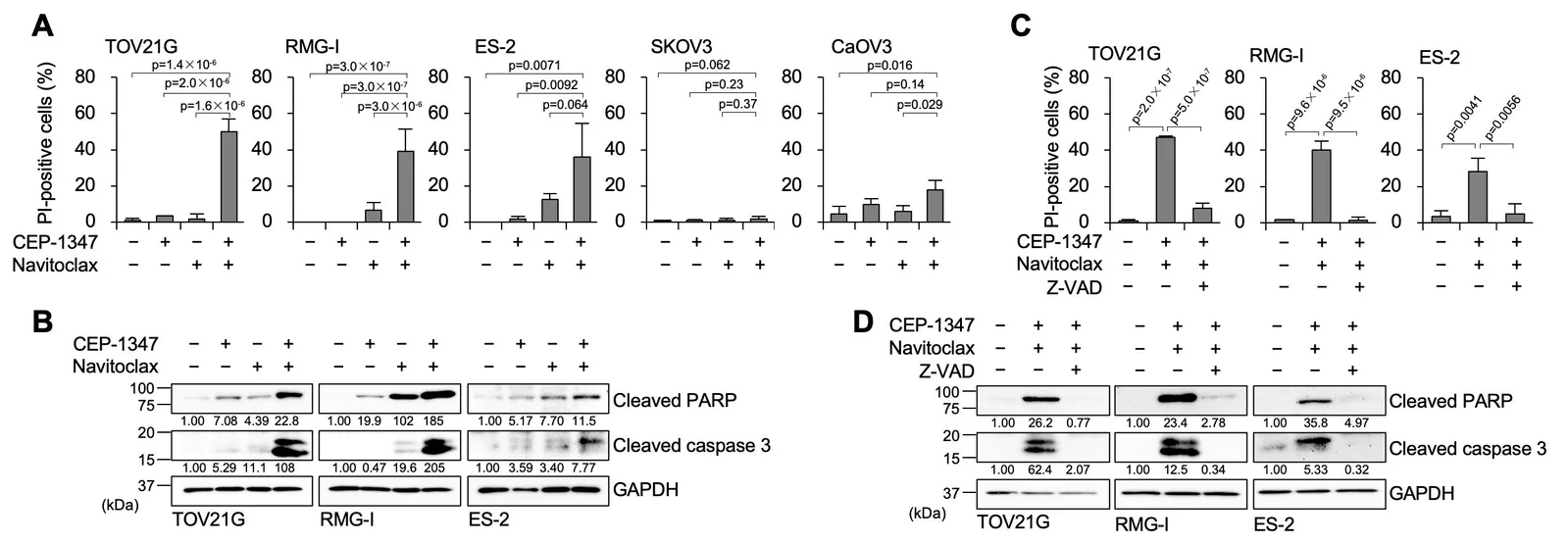
The combination of CEP-1347 and navitoclax potently induces apoptotic cell death preferentially in OCCC cells with different *TP53* gene statuses. (**A**) OCCC cells (wild-type p53: TOV21G, RMG-I, mutant p53: ES-2) and non-OCCC cells (mutant p53: SKOV3 and CaOV3) treated with 0.25 μM CEP-1347 and 0.5 μM navitoclax for 3 days were subjected to a PI uptake assay. Values represent the mean + SD of triplicate samples of a representative experiment. The total number of cells counted for each cell line under each treatment (no drugs/CEP-1347/Navitoclax/CEP-1347+Navitoclax) was 234/91/344/44 (TOV21G), 381/304/138/81 (RMG-I), 114/88/96/40 (ES-2), 528/316/550/458 (SKOV3), and 194/146/159/120 (CaOV3), respectively. Values represent the mean + SD of triplicate samples of a representative experiment. The indicated *p*-values were from a one-way ANOVA with Tukey’s post hoc test (*n* = 3). In the quantitative analysis, at least 3 fields were assessed for each well/dish. (**B**) Cells (TOV21G, RMG-I, and ES-2) were treated as described in (**A**) and subjected to a Western blot analysis. (**C**,**D**) Cells (TOV21G, RMG-I, and ES-2) pretreated with 40 μM Z-VAD-FMK for 30 min followed by 0.25 μM CEP-1347 and 0.5 μM navitoclax for 3 days (**C**) or 2 days (**D**) were subjected to the PI uptake assay (**C**) and Western blot analysis (**D**). In (**C**), the total number of cells counted for each cell line under each treatment (no drugs/CEP-1347+Navitoclax/CEP-1347+Navitoclax+Z-VAD) was 636/233/133 (TOV21G), 208/263/195 (RMG-I), and 93/97/152 (ES-2), respectively. Values represent the mean + SD of triplicate samples of a representative experiment. The indicated *p*-values were from a one-way ANOVA with Tukey’s post hoc test (*n* = 3). In the quantitative analysis, at least 3 fields were assessed for each well/dish. The numbers below the Western blot images represent relative band intensities after each band was quantified by densitometry and normalized to the GAPDH value. Representative results from three independent experiments are shown.

In contrast to HGSOC, the predominant EOC subtype in which the majority of cases harbor *TP53* mutations, OCCC exhibits a lower frequency of these mutations [15,16,17]. We previously reported that the combination of CEP-1347 and navitoclax, a BH3 mimetic targeting BCL2/BCLxL, potently induced cell death in OCCC cells with wild-type *TP53* [13]. However, the effects of this combination on OCCC cells with *TP53* mutations or on non-OCCC cells, including HGSOC cells, have yet to be elucidated. Therefore, we herein examined the cell death-inducing effects of the CEP-1347/navitoclax combination in OCCC (TOV21G, RMG-I, and ES-2) and non-OCCC (SKOV3 and CaOV3) cell lines. As previously reported [13], CEP-1347 or navitoclax alone did not cause cell death in TOV21G and RMG-I cells, harboring wild-type *TP53* [18,19], whereas their combination potently induced cell death (Figure 1A). Similarly, the combination induced cell death in ES-2 cells, one of the OCCC cell lines harboring mutant *TP53* [20] (Figure 1A). In contrast, this combination had a negligible effect or induced modest cell death in the non-OCCC cell lines harboring mutant *TP53*, SKOV3 and CaOV3 [15,21] (Figure 1A). Moreover, the combination of CEP-1347 and navitoclax activated the caspase pathway in parallel with cell death (Figure 1B), and this was suppressed by a pretreatment with Z-VAD-FMK, a pan-caspase inhibitor (Figure 1C,D). These results indicate that CEP-1347 enhanced navitoclax-induced apoptotic cell death preferentially in OCCC cells with different *TP53* gene statuses.

### 2.2. CEP-1347 Suppresses MCL1 Expression in OCCC Cells

**Figure 2:**
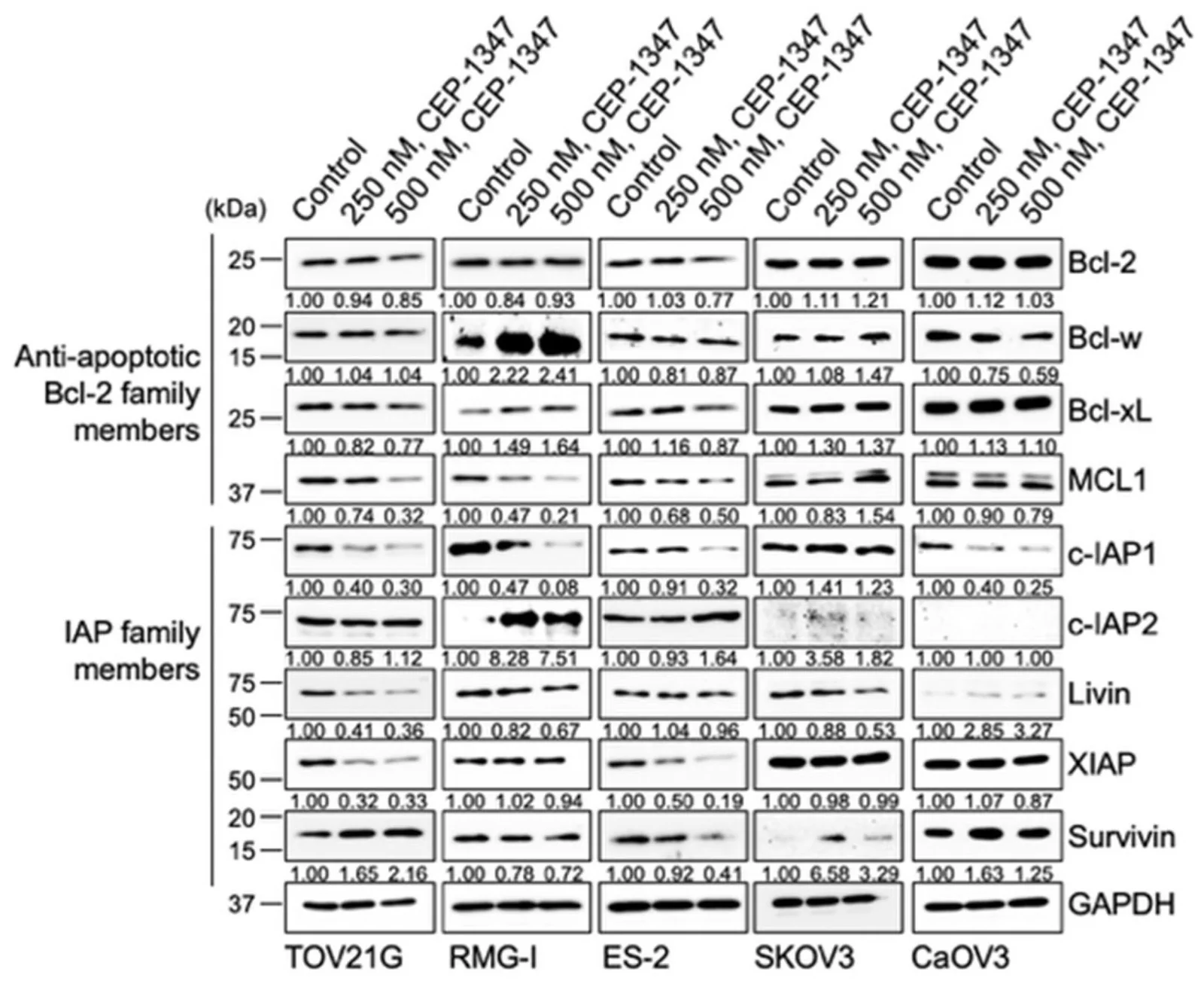
CEP-1347 inhibits the expression of MCL1 in OCCC cells. OCCC cells (TOV21G, RMG-I, and ES-2) and non-OCCC cells (SKOV3 and CaOV3) treated with CEP-1347 at the indicated concentrations for 3 days were subjected to a Western blot analysis. The numbers below the Western blot images represent relative band intensities after each band was quantified by densitometry and normalized to the GAPDH value. Representative images from two independent experiments are shown.

The above results indicate that CEP-1347 enhanced navitoclax-induced apoptotic cell death in OCCC cells with different *TP53* gene statuses. To elucidate the underlying mechanisms, we examined the effects of CEP-1347 on the expression of anti-apoptotic factors, such as members of the anti-apoptotic BCL2 and inhibitor of apoptosis (IAP) families. Across various cancer types, including ovarian cancer, tumor cells frequently overexpress anti-apoptotic factors and depend on them for their survival [22,23]. In OCCC, an analysis of a publicly available transcriptome dataset (GSE29450, https://www.ncbi.nlm.nih.gov/geo/query/acc.cgi?acc=GSE29450, accessed on 26 August 2026) revealed the higher expression of several anti-apoptotic factors than in normal tissues. In addition, functional redundancy has been reported among the anti-apoptotic BCL2 family [24,25]. Therefore, we hypothesized that anti-apoptotic factors that functionally complement BCL2/BCLxL, the targets of navitoclax, may be expressed in OCCC cells, and also that CEP-1347 may enhance sensitivity to navitoclax by targeting these factors. To confirm this hypothesis, we screened for a panel of anti-apoptotic factors with protein expression levels that were affected by CEP-1347 in the ovarian cancer cell lines used in the present study. We found that the expression levels of MCL1, a member of the anti-apoptotic BCL2 family, and c-IAP1, a member of the IAP family, decreased in a CEP-1347 dose-dependent manner in OCCC cells (Figure 2). In contrast, the expression levels of other members of the anti-apoptotic BCL2 family and IAP family were not consistently changed by the CEP-1347 treatment (Figure 2). These results suggest that CEP-1347 enhanced the sensitivity of OCCC cells to navitoclax by suppressing the anti-apoptotic factors functionally complementing the targets of navitoclax, such as c-IAP1 and MCL1.

### 2.3. CEP-1347 Enhances the Sensitivity of OCCC Cells to Navitoclax by Suppressing MCL1

**Figure 3:**
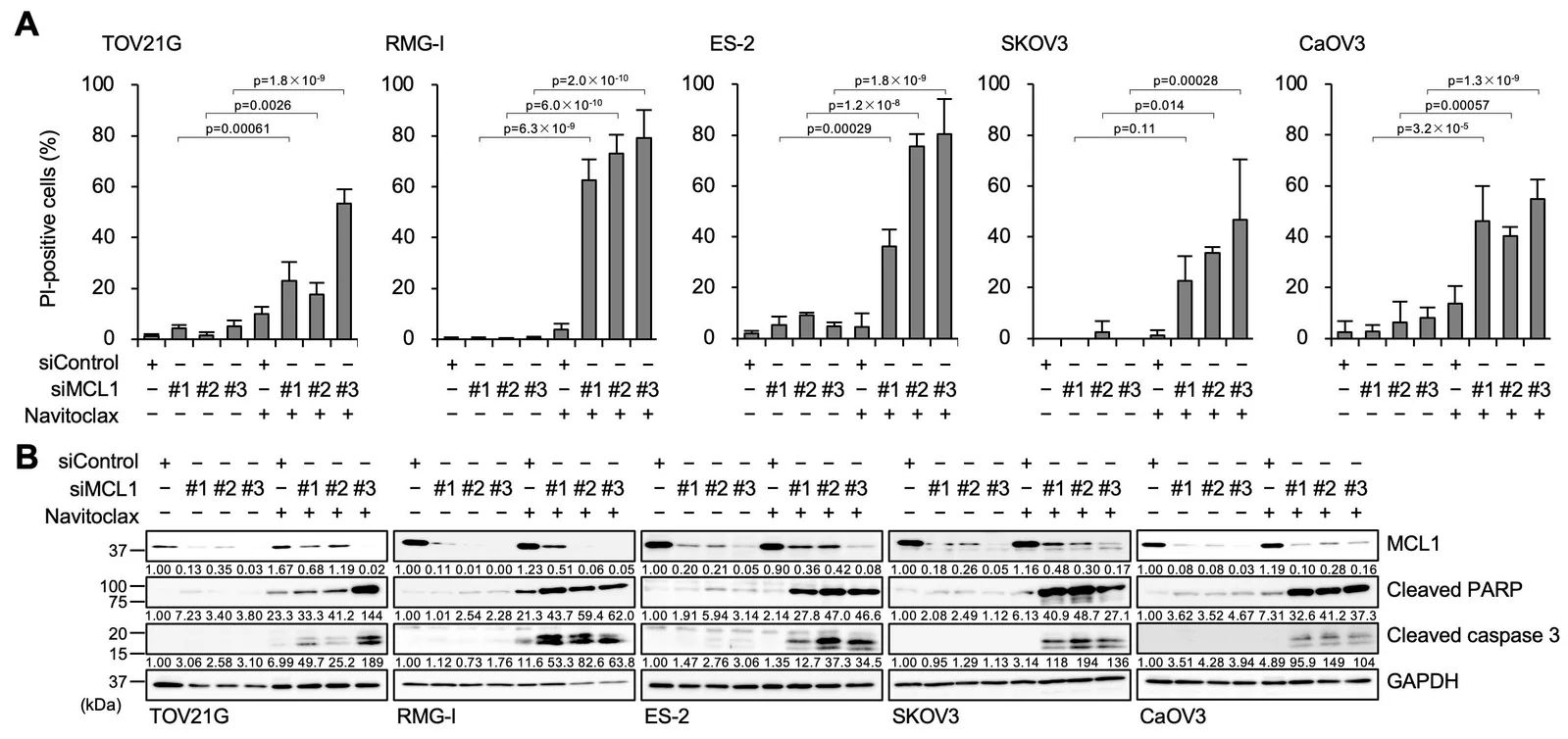
MCL1 depletion sensitizes OCCC cells to navitoclax. (**A**) OCCC cells (TOV21G, RMG-I, and ES-2) and non-OCCC cells (SKOV3 and CaOV3) transfected with control siRNA or siRNA against MCL1 (siMCL1-#1, #2, and #3) for 2 days (TOV21G) or for 1 day (RMG-1, ES-2, SKOV3 and CaOV3) and then treated with 0.5 μM navitoclax for 3 days (TOV21G) or for 1 day (RMG-1, ES-2, SKOV3 and CaOV3) were subjected to a PI uptake assay. Values represent the mean + SD of triplicate samples of a representative experiment. The total number of cells counted for each cell line under each treatment (siControl/siMCL1#1/siMCL1#2/siMCL1#3/siControl+Navitoclax/siMCL1#1+Navitoclax/siMCL1#2+Navitoclax/siMCL1#3+Navitoclax) was 416/850/664/463/446/318/404/267 (TOV21G), 574/796/686/523/525/122/176/122 (RMG-I), 567/634/574/780/444/439/145/207 (ES-2), 75/69/81/148/82/71/56/52 (SKOV3), and 96/81/74/82/105/222/161/91 (CaOV3), respectively. The indicated *p*-values were from a one-way ANOVA with Tukey’s post hoc test (*n* = 3). In the quantitative analysis, at least 3 fields were assessed for each well/dish. Representative results from three independent experiments are shown. (**B**) Cells transfected as in (A) and then treated with 0.5 μM navitoclax for 48 h (TOV21G) or for 12 h (RMG-1, ES-2, SKOV3 and CaOV3) were subjected to a Western blot analysis. The numbers below the Western blot images represent relative band intensities after each band was quantified by densitometry and normalized to the GAPDH value. Representative images of three independent biological replicates.

Since the CEP-1347 treatment decreased the expression levels of c-IAP1 and MCL1, we hypothesized that the CEP-1347-induced enhancements in the sensitivity of OCCC cells to navitoclax may be due to the suppression of these anti-apoptotic factors. Therefore, we investigated whether inhibiting the expression of these proteins was sufficient to sensitize OCCC cells to navitoclax. To achieve this, we knocked down c-IAP1 or MCL1 in OCCC cells, and subsequently investigated whether navitoclax induced cell death. The depletion of c-IAP1 did not consistently enhance navitoclax sensitivity in OCCC cells (Supplementary Figure S1), whereas the depletion of MCL1 markedly enhanced navitoclax-induced cell death (Figure 3A). Furthermore, the addition of navitoclax after the knockdown of MCL1 activated the caspase pathway in parallel with cell death (Figure 3B), and the induction of cell death and caspase activation occurred regardless of the *TP53* gene status (Figure 3A,B). The depletion of MCL1 potently enhanced the sensitivity to navitoclax, even in non-OCCC cell lines, in which the effects of enhancing sensitivity to navitoclax and decreasing MCL1 expression by CEP-1347 were weak (Figure 3A,B).

**Figure 4:**
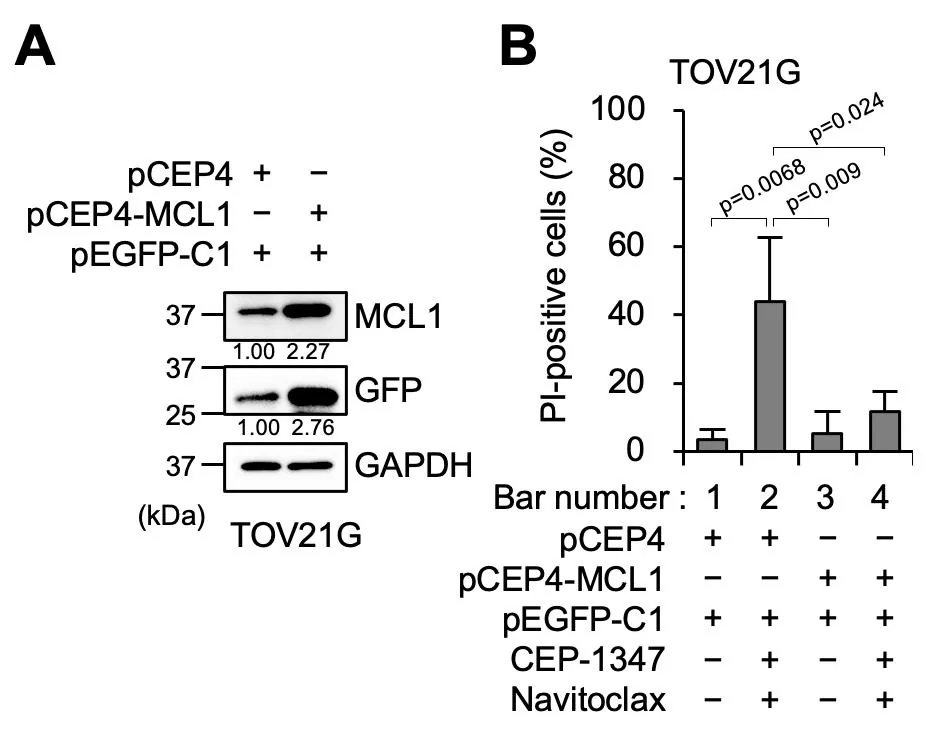
Overexpression of MCL1 suppresses CEP-1347/navitoclax-induced cell death. (**A**) TOV21G cells were transfected with the plasmids indicated. One day later, cells were subjected to a Western blot analysis. The numbers below the Western blot images represent relative band intensities after each band was quantified by densitometry and normalized to the GAPDH value. (**B**) TOV21G cells were transfected as described in (**A**). One day later, cells were treated with 0.25 μM CEP-1347 and 0.5 μM navitoclax for 3 days and then subjected to a PI uptake assay to assess the percentage of PI-positive GFP-expressing cells. Values represent the mean + SD of triplicate samples of a representative experiment. The total number of cells counted under each treatment (Bar1/Bar2/Bar3/Bar4) was 47/53/80/66. The indicated *p*-values were from a one-way ANOVA with Tukey’s post hoc test (*n* = 3). In the quantitative analysis, at least 3 fields were assessed for each well/dish. Representative results from three independent experiments are shown.

These results demonstrate that the genetic suppression of MCL1 was sufficient to enhance sensitivity to navitoclax in both OCCC and non-OCCC cells. We also investigated whether inhibiting MCL1 expression in OCCC cells was required for CEP-1347/navitoclax-induced cell death. To achieve this, we examined the effects of the forced overexpression of MCL1 on cell death induced by the CEP-1347/navitoclax combination in OCCC cells. Overexpression of MCL1 inhibited combination treatment-induced cell death (Figure 4). These results suggest that the enhancement in navitoclax sensitivity in OCCC cells induced by CEP-1347 was at least in part mediated by the downregulated expression of MCL1.

### 2.4. CEP-1347, Which Suppressed MCL1 Expression Preferentially in OCCC Cells, Enhances Navitoclax Sensitivity in OCCC Cells Without Any Toxicity in Normal Cells, Unlike S63845, an MCL1-Targeting BH3 Mimetic

**Figure 5:**
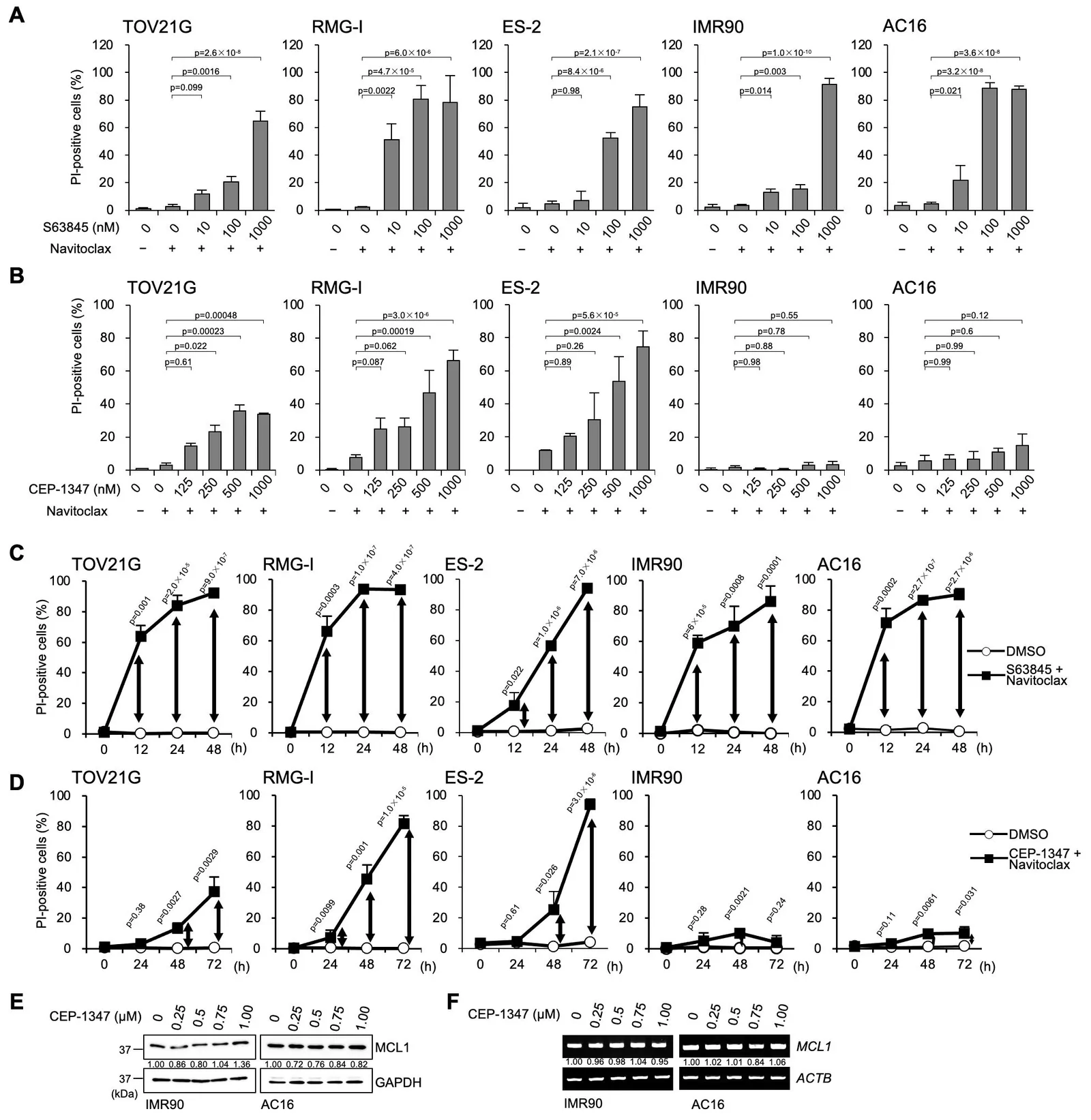
CEP-1347 sensitizes OCCC cells to navitoclax without causing toxicity to normal cells in contrast to S63845, an MCL1 targeting BH3 mimetic. (**A**) OCCC cells (TOV21G, RMG-I, and ES-2), the human normal fibroblast cell line IMR90, and the human cardiomyocyte cell line AC16 treated with 0.5 μM navitoclax and the indicated concentrations of S63845 for 1 day were subjected to a PI uptake assay. The total number of cells counted for each cell line under each treatment (no drugs/Navitoclax/10 nM S63845+Navitoclax/100 nM S63845+Navitoclax/1000 nM S63845+Navitoclax) was 379/536/493/415/286 (TOV21G), 450/386/77/60/65 (RMG-I), 51/97/50/92/184 (ES-2), 185/249/240/294/147 (IMR90), and 208/218/166/478/607 (AC16), respectively. (**B**) Cells treated with 0.5 μM navitoclax and the indicated concentrations of CEP-1347 for 3 days were subjected to the PI uptake assay. The total number of cells counted for each cell line under each treatment (no drugs/Navitoclax/125 nM CEP-1347+Navitoclax/250 nM CEP-1347+Navitoclax/500 nM CEP-1347+Navitoclax/1000 nM CEP-1347+Navitoclax) was 436/467/204/137/147/209 (TOV21G), 286/171/144/222/284/341 (RMG-I), 59/85/54/191/94/144 (ES-2), 250/327/395/326/240/235 (IMR90), and 238/217/250/251/255/154 (AC16), respectively. Values represent the mean + SD of triplicate samples of a representative experiment. The indicated *p*-values were from a one-way ANOVA with Tukey’s post hoc test (*n* = 3). In the quantitative analysis, at least 3 fields were assessed for each well/dish. (**C**) Cells treated with 0.5 μM navitoclax and 1 μM S63845 for the indicated times were subjected to the PI uptake assay. The total number of cells counted for each cell line under each condition (0 h/12 h/24 h/48 h) was 241/338/460/377 (TOV21G, DMSO), 365/690/655/372 (RMG-I, DMSO), 372/558/776/514 (ES-2, DMSO), 61/97/93/365 (IMR90, DMSO), and 325/428/675/367 (AC16, DMSO), 196/191/148/160 (TOV21G, S63845), 479/229/219/410 (RMG-I, S63845), 455/322/305/353 (ES-2, S63845), 138/154/81/48 (IMR90, S63845), and 165/346/458/248 (AC16, S63845), respectively. (**D**) Cells treated with 0.5 μM navitoclax and 0.5 μM CEP-1347 for the indicated times were subjected to the PI uptake assay. The total number of cells counted for each cell line under each treatment (0 h/24 h/48 h/72 h) was 168/232/289/821 (TOV21G, DMSO), 304/456/413/753 (RMG-I, DMSO), 147/276/786/700 (ES-2, DMSO), 70/96/198/190 (IMR90, DMSO), and 286/275/469/673 (AC16, DMSO), 142/106/117/135 (TOV21G, CEP-1347), 522/344/162/133 (RMG-I, CEP-1347), 233/229/176/159 (ES-2, CEP-1347), 171/73/208/97 (IMR90, CEP-1347), and 302/185/219/140 (AC16, CEP-1347), respectively. Values represent the mean + SD of triplicate samples of a representative experiment. Exact *p*-values shown for each comparison (indicated with a double-headed arrow) between the DMSO-treated group and the CEP-1347+Navitoclax-treated group at the same concentrations were from the two-tailed Student’s *t*-test (*n* = 3). In the quantitative analysis, at least 3 fields were assessed for each well/dish. (**E**,**F**) IMR90 and AC16 cells treated with the indicated concentrations of CEP-1347 for 3 days were subjected to a Western blot analysis (**E**) and semi-quantitative RT-PCR analysis (**F**). The numbers below the Western blot and semi-quantitative RT-PCR images represent relative band intensities after each band was quantified by densitometry and normalized to the GAPDH or *ACTB* value. Representative images of two independent biological replicates.

The above results suggest that the enhancement in navitoclax sensitivity in OCCC cells by CEP-1347 was due to the inhibition of MCL1 expression. A previous study reported that MCL1 regulated the sensitivity of several cancer cell lines to navitoclax [26,27], and MCL1-targeting BH3 mimetics have attracted attention as promising therapeutic anticancer agents; however, their effects in OCCC remain unclear. Therefore, we examined the effects of the combination of S63845, a representative MCL1-targeting BH3 mimetic, with navitoclax on OCCC cells. Consistent with the results obtained following the knockdown of MCL1, the S63845 treatment enhanced the sensitivity of OCCC cell lines to navitoclax (Figure 5A). However, the combination of S63845 and navitoclax also strongly induced cell death in the human normal fibroblast cell line IMR90 (Figure 5A). The inhibition of MCL1 using BH3 mimetics targeting MCL1, including S63845, has been shown to potently induce cardiomyotoxicity [28,29]. In addition, the combination of S63845 and navitoclax was found to have very poor tolerability [30]. Therefore, we performed the same cytotoxicity analysis using not only IMR90 cells, but also AC16 cells, a normal human adult ventricular cardiomyocyte cell line that is frequently used in toxicity analyses in MCL1 inhibition studies [31]. The combination of S63845 and navitoclax potently induced cell death in both IMR90 and AC16 cells (Figure 5A). In contrast, the combination of CEP-1347 and navitoclax induced cell death preferentially in OCCC cells while causing only minimal cell death in normal cells, such as IMR90 fibroblasts and AC16 cardiomyocytes (Figure 5B). Consistent with these results, a time-course analysis revealed that the combination treatment of S63845 with navitoclax potently induced cell death in all cell lines used in this study, including normal cells, within 24 h, whereas CEP-1347 promoted navitoclax-induced cell death preferentially in OCCC cells while minimizing cell death in normal cells (Figure 5C,D). To elucidate the mechanisms underlying the OCCC-selective enhancement in navitoclax sensitivity, we examined the effects of CEP-1347 on MCL1 expression in IMR90 and AC16 cells. We found that CEP-1347 exerted negligible or minimal effects on MCL1 protein and mRNA expression levels in IMR90 and AC16 cells (Figure 5E,F). These results suggest that CEP-1347 preferentially enhanced navitoclax sensitivity in OCCC cells by downregulating MCL1 without any effects on the viability of normal cells, such as IMR90 and AC16, in contrast to the MCL1-targeting BH3 mimetic, S63845.

## 3. Discussion

OCCC is less sensitive to conventional chemotherapy than HGSOC, which is the predominant subtype of EOC, and current treatment strategies for OCCC are often extrapolated from clinical trial data largely derived from patients with HGSOC. Therefore, there remains a substantial need for effective systemic therapies specifically tailored to OCCC [2,4]. We previously reported that CEP-1347, for which safety data on its long-term administration in humans are available, enhances the sensitivity of OCCC cells to navitoclax and also that the combination of CEP-1347 and navitoclax potently induced cell death in OCCC cells [13]. Therefore, a more detailed understanding of the molecular mechanisms underlying the CEP-1347-mediated sensitization of OCCC cells to navitoclax is important for the future development of safe and effective CEP-1347-based therapeutic strategies for ovarian cancer. In the present study, we demonstrated that CEP-1347 preferentially sensitized OCCC cells over non-OCCC cells to navitoclax, and also that this difference was not necessarily attributable to differences in the *TP53* gene status. In addition, CEP-1347 preferentially inhibited MCL1 expression in OCCC cells over non-OCCC cells, and the present results indicate that the downregulation of MCL1 contributed, at least in part, to the CEP-1347-mediated sensitization of OCCC cells to navitoclax. Furthermore, the genetic depletion of MCL1 enhanced sensitivity to navitoclax not only in OCCC cells, but also in non-OCCC cells. Collectively, these results suggest that MCL1 expression is an important factor contributing to navitoclax sensitivity in both OCCC and non-OCCC cells and also that the preferential downregulation of MCL1 by CEP-1347 in OCCC cells underlies, at least in part, the preferential sensitization of OCCC cells to navitoclax. Importantly, the present results showed that CEP-1347 preferentially inhibited MCL1 expression in OCCC cells at concentrations that had negligible or no effects on MCL1 expression in IMR90 normal fibroblasts and AC16 normal cardiomyocytes. In contrast, S63845, an MCL1-targeting BH3 mimetic, sensitized these normal cells, including cardiomyocytes and fibroblasts, as well as OCCC cells to navitoclax. These in vitro results are consistent with previous findings on the toxicity associated with MCL1-targeting BH3 mimetics [28,29,30]. Therefore, CEP-1347, which downregulated MCL1 and enhanced navitoclax sensitivity preferentially in OCCC cells, has potential as a safer and effective alternative to MCL1-targeting BH3 mimetics for the treatment of OCCC.

Although the present study showed that the CEP-1347-induced downregulation of MCL1 and enhancement in navitoclax sensitivity occurred preferentially in OCCC cells, the molecular mechanisms underlying these preferential effects and selectivity remain unclear. CEP-1347 may target differences in the mechanisms that maintain MCL1 expression between OCCC cells and non-OCCC cells or normal cells, such as IMR90 and AC16 cells. One potential factor underlying these differences is the *TP53* status because OCCC, unlike non-OCCC, frequently retains wild-type *TP53* [15,16,17]. In the non-OCCC cell lines used in the present study, which reportedly harbor mutant *TP53*, CEP-1347 exerted only modest effects on MCL1 expression and navitoclax sensitivity. However, CEP-1347 decreased MCL1 expression in the OCCC cell line ES-2, which harbors mutant *TP53*, but exerted negligible or no effects on MCL1 expression in IMR90 normal human fibroblasts or AC16 normal human adult ventricular cardiomyocytes. AC16 cells are frequently used as a control model for evaluating the cardiotoxicity associated with MCL1 inhibition [31]. Since AC16 cells were established by SV40-mediated transformation, the function of p53 is also likely to be secondarily suppressed in these cells [32]. Furthermore, in our previous study, CEP-1347 enhanced sensitivity to navitoclax in glioma stem cells retaining wild-type *TP53*, but had negligible or only minimal effects on MCL1 expression in these cells [14]. Collectively, these findings and the present results suggest that p53 is unlikely to play a major role in the CEP-1347-mediated downregulation of MCL1.

Other potential mechanisms underlying the preferential inhibition of MCL1 expression by CEP-1347 in OCCC cells may involve activating mutations in PIK3CA or loss-of-function mutations in *ARID1A*, both of which are characteristic molecular changes in OCCC. Several E3 ubiquitin ligases, including Mule, β-TrCP, and FBXW7, have been shown to regulate MCL1 protein levels [33,34,35]. The phosphorylation of MCL1 by GSK3β is known to be required for its ubiquitination mediated by β-TrCP and FBXW7 [34,36,37]. Since activating mutations in *PIK3CA* occur in approximately 20% of OCCC cases [38], activation of the canonical PI3K–Akt pathway may inactivate GSK3β in these tumors [39]. Furthermore, navitoclax has been reported to promote MCL1 stabilization in a hepatocellular carcinoma cell line by activating Akt and thereby inactivating GSK3β [40]. In the present study, we observed a modest increase in MCL1 expression following the navitoclax treatment in TOV21G cells. Taken together, these findings and the present results suggest that CEP-1347 suppressed MCL1 expression in OCCC cells by activating GSK3β through the inhibition of the PI3K–Akt pathway. However, crosstalk between CEP-1347 and the GSK3β pathway has not been reported to date. Moreover, among the OCCC cell lines used in this study, ES-2 and RMG-I are known to harbor wild-type *PIK3CA* [41]. Therefore, additional signaling pathways may be involved, and further investigations are warranted. Another possible mechanism involves deubiquitinating enzymes that regulate MCL1 protein stability, including USP9X and USP13 [42,43]. USP9X was previously suggested to be upregulated in association with mutations in *ARID1A* [44], a component of the SWI/SNF complex that is mutated in approximately 50% of OCCC cases [45]. Therefore, CEP-1347 may destabilize MCL1 by suppressing USP9X in *ARID1A*-mutant OCCC cells. However, although TOV21G cells are known to harbor mutant *ARID1A*, RMG-I and ES-2 cells reportedly retain wild-type *ARID1A* [46]. Since the ARID1A–USP9X axis alone cannot fully explain these findings, multiple factors appear to be involved in the CEP-1347-mediated reduction in MCL1 expression, and thus further investigations are required.

MCL1 is a well-established factor contributing to sensitivity not only to navitoclax, but also to BCLxL inhibitors, such as ABT-737 [26,27,47,48]. In addition, sensitivity to navitoclax, a BCL2/BCLxL inhibitor known for its senolytic activity, may be enhanced by cellular senescence as well as low MCL1 expression levels [49]. Based on these findings, the inhibition of MCL1 has attracted increasing attention in cancer therapeutic strategies involving BH3 mimetics. However, despite accumulating evidence supporting the inhibition of MCL1 as a potentially effective anticancer strategy, MCL1 inhibitors, including S63845, a representative MCL1-selective BH3 mimetic, have not yet reached the clinical application stage because of concerns regarding their potent toxicity [28,29,30]. Given the potential toxicity associated with the inhibition of MCL1, we compared the cytotoxic effects of CEP-1347 and S63845 in two human normal cell lines and OCCC cells. S63845 enhanced navitoclax sensitivity in normal cells, whereas CEP-1347 did not. Furthermore, an analysis of the relationship between S63845 concentrations and cell death induced by its combination with navitoclax revealed that S63845 sensitized normal cells to navitoclax-induced cell death to a similar extent as OCCC cells. Therefore, the combination of an MCL1-targeting BH3 mimetic with navitoclax appears to have limited selectivity between OCCC and normal cells, whereas the combination of CEP-1347 and navitoclax exhibits greater selectivity for OCCC cells, and thus has potential as a combination strategy. However, since the present study was limited to in vitro experiments, further evaluations in preclinical models are required. An advantage of CEP-1347 with respect to its potential clinical application in cancer therapy is that information regarding its safety and pharmacokinetics in humans is already available. A previous pharmacokinetic study on humans reported that the administration of CEP-1347 at 50 mg twice daily resulted in a mean maximum plasma concentration of approximately 700 nM [5], and this dose was tolerated for up to 24 months [7]. Based on these findings, we tested CEP-1347 at concentrations up to 1000 nM in the present study and found that it did not enhance navitoclax sensitivity in normal human fibroblasts. In addition, given the potential cardiotoxicity associated with MCL1-targeting BH3 mimetics, we examined AC16 normal human adult ventricular cardiomyocytes and found that CEP-1347 only slightly enhanced navitoclax sensitivity in these cells. In contrast, CEP-1347 markedly enhanced navitoclax sensitivity in OCCC cell lines, even at concentrations <500 nM, suggesting that CEP-1347 could provide a wider therapeutic window than S63845. Therefore, CEP-1347, as a potentially safe and effective alternative to MCL1-targeting BH3 mimetics for combination with navitoclax, warrants the further evaluation of its combination with navitoclax in preclinical animal models. Given the availability of clinical safety data for CEP-1347, if favorable preclinical results are obtained, this may lay the groundwork for further investigations into the clinical feasibility of this combination therapy for patients with OCCC.

In conclusion, we herein demonstrate that CEP-1347, for which long-term safety data in humans are available, preferentially enhanced the sensitivity of OCCC cells to navitoclax by downregulating MCL1 expression. Furthermore, in comparisons with MCL1 inhibition by BH3 mimetics, CEP-1347 preferentially sensitized OCCC cells to navitoclax with minimal effects on the viability of IMR90 normal fibroblasts and AC16 normal cardiomyocytes. These results strongly suggest the potential of the combination of CEP-1347 and navitoclax as a novel therapeutic strategy for OCCC, for which substantial unmet medical needs remain.

## 4. Materials and Methods

### 4.1. Antibodies and Reagents

Antibodies against cleaved PARP (#9541), cleaved caspase 3 (#9664), GAPDH (#5174), Bcl-2 (#15071), Bcl-xL (#2764), Mcl-1 (#39224), c-IAP1 (#7065), c-IAP2 (#3130), Livin (#5471), XIAP (#2045), and Survivin (#2808) were purchased from Cell Signaling Technology, Inc. (Beverly, MA, USA). An antibody against GFP (sc-9996) was from Santa Cruz Biotechnology, Inc. (Dallas, TX, USA). An antibody against BCL2L2/Bcl-w (16026-1-AP) was purchased from ProteinTech Group, Inc. (Rosemont, IL, USA). Horseradish peroxidase (HRP)-conjugated rabbit and mouse secondary antibodies were from Jackson ImmunoResearch, Inc. (West Grove, PA, USA). CEP-1347 was obtained from Bio-Techne (Minneapolis, MN, USA). Navitoclax (ABT-263) was supplied by Chemscene (Monmouth Junction, NJ, USA). Z-VAD-FMK (3188-v) was purchased from Peptide Institute, Inc. (Osaka, Japan). S63845 was from Selleck Chemicals (Houston, TX, USA). CEP-1347, Navitoclax, Z-VAD-FMK, and S63845 were dissolved in DMSO to prepare 0.5, 0.5, 20, and 1 mM stock solutions, respectively.

### 4.2. Cell Culture

TOV21G (CRL-3577), SKOV3 (HTB-77), CaOV3 (HTB-75), and AC16 (CRL-3568) cells were obtained from the American Type Culture Collection (Manassas, VA, USA). IMR-90 cells (JCRB9054) were purchased from the Japanese Collection of Research Bioresources Cell Bank (Osaka, Japan). RMG-I cells were kindly provided by Dr. S. Nozawa and Dr. D. Aoki (Keio University, Japan), and ES-2 cells by Dr. Yaegashi (Department of Gynecology and Obstetrics, Tohoku University Graduate School of Medicine, Japan) [50]. TOV21G, RMG-I, ES-2, SKOV3, and AC16 cells were maintained in DMEM/F-12 supplemented with 10% fetal bovine serum (FBS: Thermo Fisher Scientific, Waltham, MA, USA), while CaOV3 and IMR90 cells were maintained in DMEM supplemented with 10% FBS. All IMR90 experiments were performed using cells with a low passage number (<8).

### 4.3. Propidium Iodide (PI) Uptake Assay

The PI uptake assay was performed to assess the percentage of dead cells as previously described [14,51]. Cells were incubated in situ with PI (1 μg/mL) and Hoechst 33342 (10 μg/mL) at 37 °C for 5 min in a CO_2_ incubator to stain dead cells and cell nuclei, respectively. Fluorescent images were obtained using a fluorescence microscope (CKX53; EVIDENT, Tokyo, Japan) equipped with an iPhone 7 (Apple, Cupertino, CA, USA) and scored using ImageJ software (version 1.53k, National Institutes of Health, Bethesda, MD, USA; (https://imagej.net/ij/, accessed on 30 August 2026). In the quantitative analysis, at least 3 randomly selected fields were assessed for each condition.

### 4.4. Western Blot Analysis

A Western blot analysis was conducted as previously described [51,52]. Cells were harvested, washed with ice-cold PBS, and then lysed in RIPA buffer (10 mM Tris/HCl (pH 7.4), 0.1% sodium deoxycholate, 0.1% sodium dodecyl sulfate (SDS), 1% Nonidet P-40, 150 mM NaCl, 1 mM EDTA, 10 mM sodium fluoride, 1.5 mM sodium orthovanadate, 10 mM sodium pyrophosphate, and protease inhibitor cocktail set III (FUJIFILM Wako Chemicals, Osaka, Japan)). The lysate was immediately mixed with the same volume of 2 × Laemmli buffer (125 mM Tris/HCl (pH 6.8), 4% SDS, and 10% glycerol) and boiled at 95 °C for 10 min. Samples with protein concentrations measured using a BCA protein assay kit (Thermo Fisher Scientific) were separated by SDS/polyacrylamide gel electrophoresis and transferred to polyvinylidene difluoride membranes (Immobilon-P, Merck KGaA, Darmstadt, Germany). The membranes were reacted with primary antibodies followed by appropriate HRP-conjugated secondary antibodies, and then detected using Immobilon Western Chemiluminescent HRP Substrate (Merck KGaA). Immunoreactive bands were detected using a ChemiDoc Touch device (Bio-Rad Laboratories, Inc., Hercules, CA, USA). The quantification of bands on the membranes was performed by densitometry using ImageJ software (version 1.53a) (https://imagej.net/ij/).

### 4.5. Reverse Transcription (RT) Analysis

A semi-quantitative RT-PCR analysis was conducted as previously described [11]. Total RNA was extracted from cells using Trizol (Thermo Fisher Scientific), and 1 μg of total RNA was reverse transcribed using the PrimeScript RT Reagent Kit (Takara Bio Inc., Shiga, Japan) according to the manufacturer’s protocol. The target genes were amplified with Quick Taq HS DyeMix (Toyobo Co., Ltd., Osaka, Japan) using the primer sets listed below: *MCL1* (Fw: 5′-GAGTTGGTCGGGGAATCTGG-3′, Rv: 5′-TCCTGCCCCAGTTTGTTACG-3′), and *ACTB* (Fw: 5′-CCCATGCCATCCTGCGTCTG-3′, Rv: 5′-CGTCATACTCCTGCTTGCTG-3′).

### 4.6. Transfection of siRNA or Plasmids

AllStars Negative Control siRNA (QIAGEN, Venlo, The Netherlands) or siRNAs against MCL1 (#1: HSS181041, #2: HSS181042, #3: HSS181043) and c-IAP1 (#1: HSS100558, #2: HSS179382, #3: HSS179383) (Thermo Fisher Scientific) were transfected using Lipofectamine RNAiMAX (Thermo Fisher Scientific) in accordance with the manufacturer’s instructions. Human MCL1 (NM_021965) was subcloned into the pCEP4 plasmid (Thermo Fisher Scientific, V04450). This plasmid was confirmed by DNA sequencing. The pCEP4-MCL1 plasmid and the pCEP4 and pEGFP-C1 control plasmids (GenBank accession #U55763; Takara Bio Inc., Shiga, Japan) were transiently transfected using Lipofectamine 2000 (Thermo Fisher Scientific) in accordance with the manufacturer’s instructions.

### 4.7. Data Reproducibility and Statistical Analysis

The PI uptake assay, Western blotting, and semi-quantitative RT-PCR analyses were repeated at least twice with similar results, and one set of representative data is presented. Data analyses were performed using the software Microsoft Excel (Version 2609, Redmond, Washington, WA, USA) for the Student’s two-tailed *t*-test or EZR (Version 1.70, Saitama Medical Center, Jichi Medical University), a graphical user interface for R [53], for one-way ANOVA followed by Tukey’s post hoc test. Results are expressed as the mean and standard deviation (SD). The significance of differences was assessed using the Student’s two-tailed *t*-test for comparisons of two groups. A one-way ANOVA followed by Tukey’s post hoc test was applied for multiple comparisons. All cell experiments were performed using at least two biological replicates, with similar results, and one representative dataset is presented.

## Data Availability

All data are contained in this article and there are no repository data.

## Supplementary Materials

The following supporting information can be downloaded at https://www.mdpi.com/article/10.3390/ijms27209261/s1.
