# Supplementary material for: CEP-1347 Enhances the Sensitivity of Ovarian Clear Cell Carcinoma Cells to Navitoclax by Downregulating MCL1
Source: Int J Mol Sci. 2026 Oct 19;27(20):9261. doi: 10.3390/ijms27209261 (PMC13705146; doi:10.3390/ijms27209261)
Supplement: Supplementary file 1 [file ijms-27-09261-s001.zip › ijms-4585323-supplementary.pdf]

Supplementary Figure S1

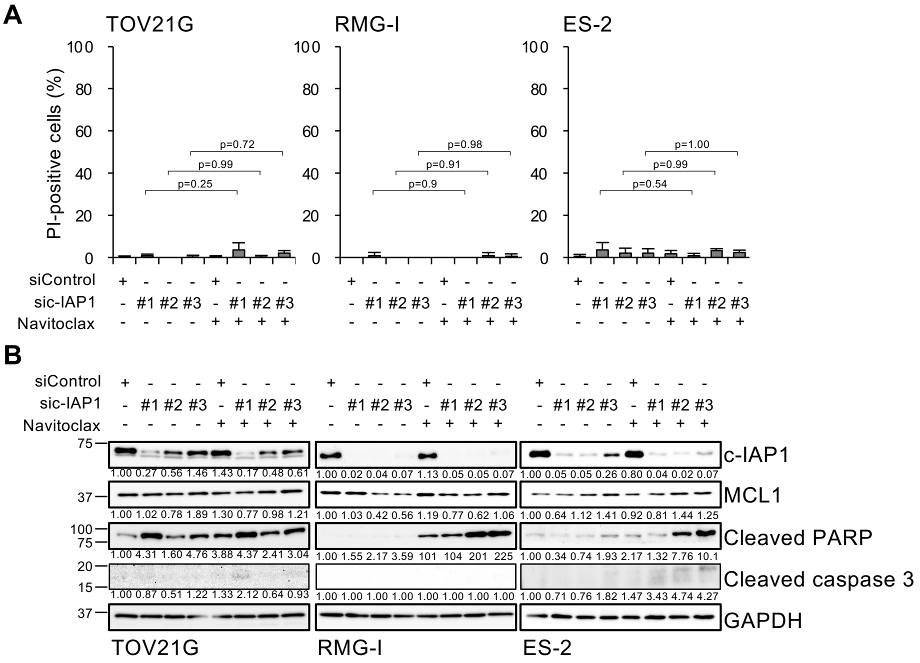

Supplementary Figure S1. Effects of c-IAP1 depletion on the navitoclax sensitivity of OCCC cells. (A, B) Cells (TOV21G, RMG-I, and ES-2) were transfected with control siRNA or siRNA against c-IAP1 (sic-IAP1-#1, #2, and #3). One day later, cells treated with 0.5 μM navitoclax for 3 days (TOV21G), 2 days (RMG-I), or 1 day (ES-2) were subjected to a PI uptake assay (A) and Western blot analysis (B). The total numbers of cells counted for each cell line under each treatment (siControl/siMCL1#1/siMCL1#2/siMCL1#3/siControl+Navitoclax/siMCL1#1+Navitoclax/siMCL1#2+Navitoclax/siMCL1#3+ Navitoclax) were 335/327/441/241/367/163/315/300 (TOV21G), 96/112/149/167/108/87/100/130 (RMG-I), and 164/127/171/201/120/134/121/201 (ES-2), respectively.

Supplementary Figure S2

(A) Figure 1A

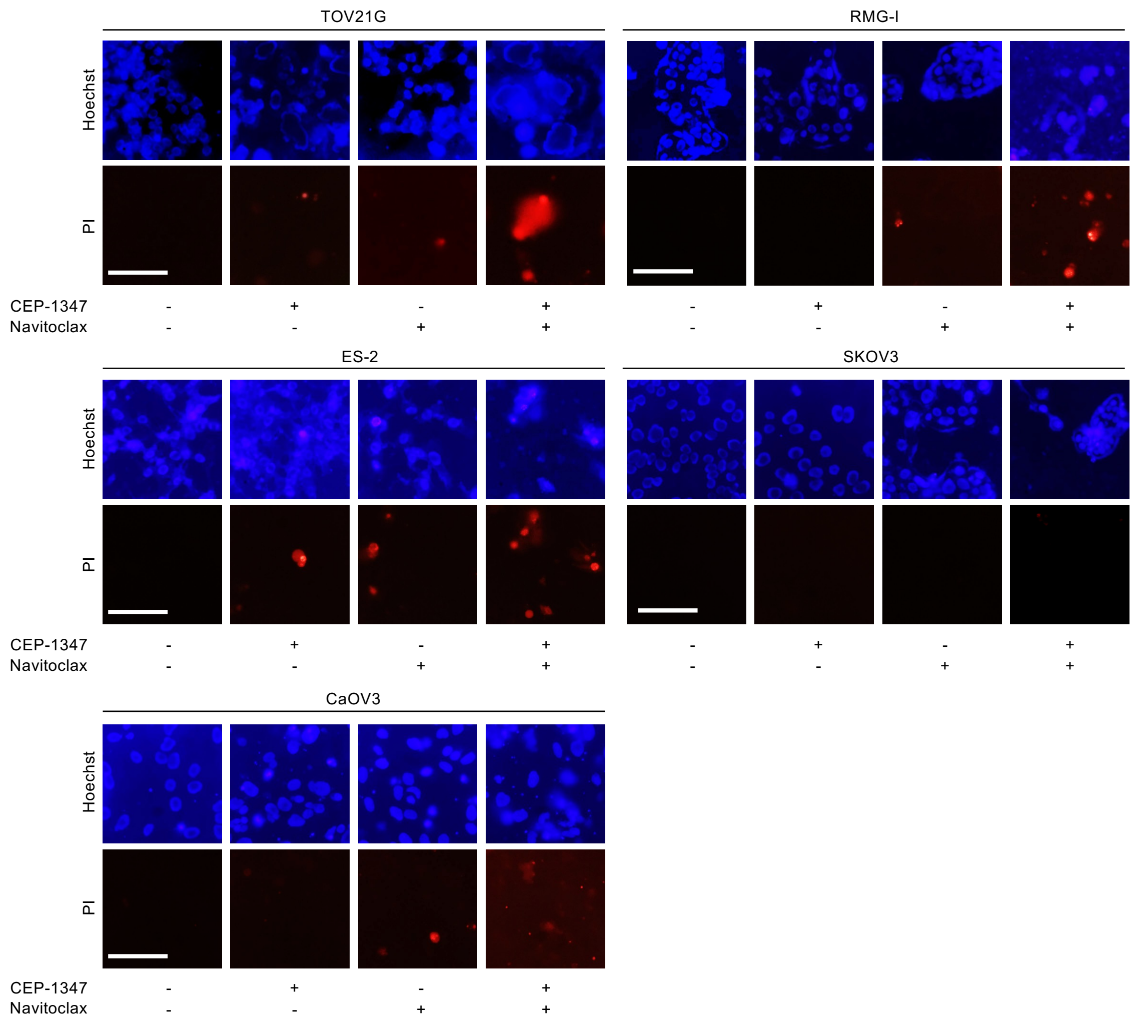

(B) Figure 1C

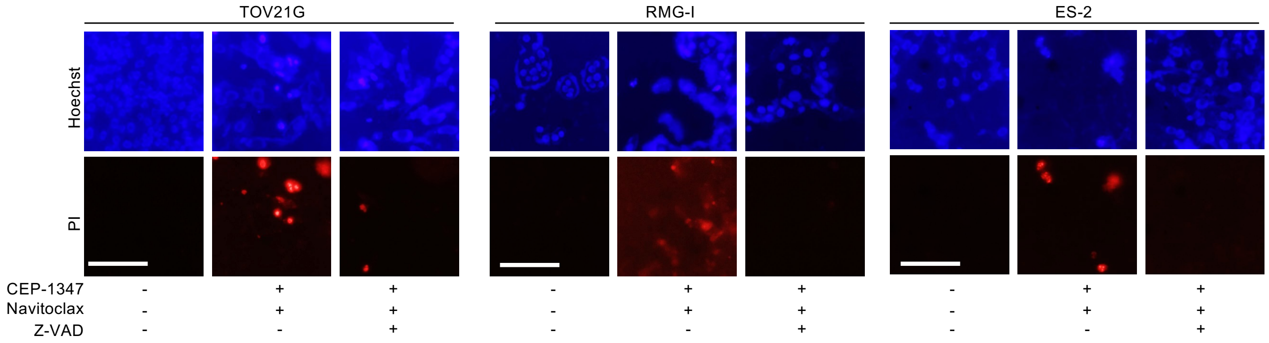

Supplementary Figure S2 (continued)

(C) Figure 3A

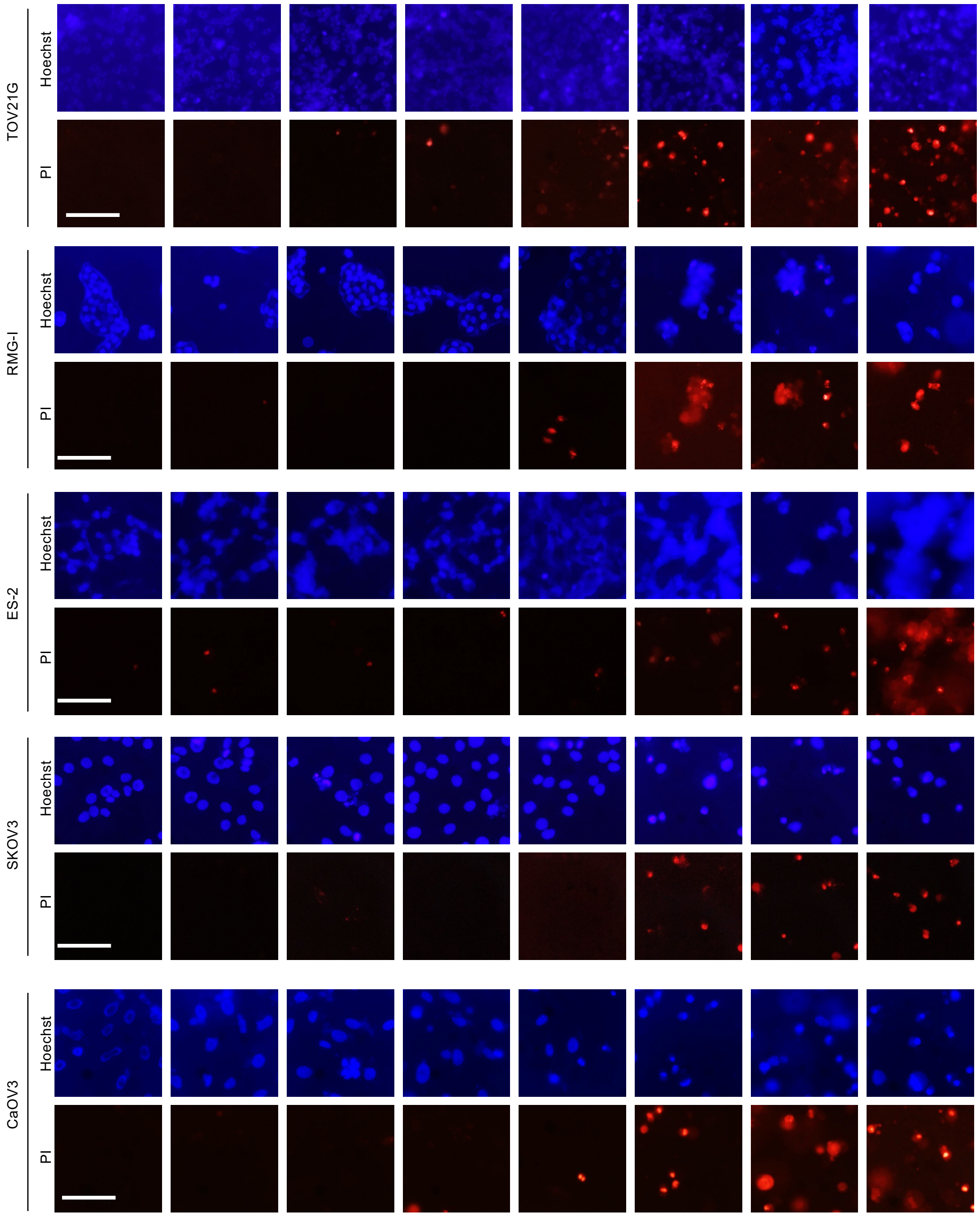

siControl	+	-	-	-	+	-	-	-
siMCL1	-	#1	#2	#3	-	#1	#2	#3
Navitoclax	-	-	-	-	+	+	+	+

(D) Figure 4B

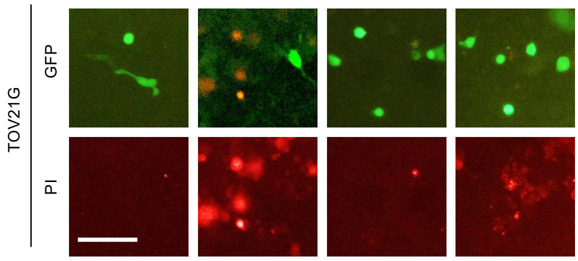

Bar number	1	2	3	4
pCEP4	+	+	-	-
pCEP4-MCL1	-	-	+	+
pEGFP-C1	+	+	+	+
CEP-1347	-	+	-	+
Navitoclax	-	+	-	+

Supplementary Figure S2 (continued)

(E) Figure 5A

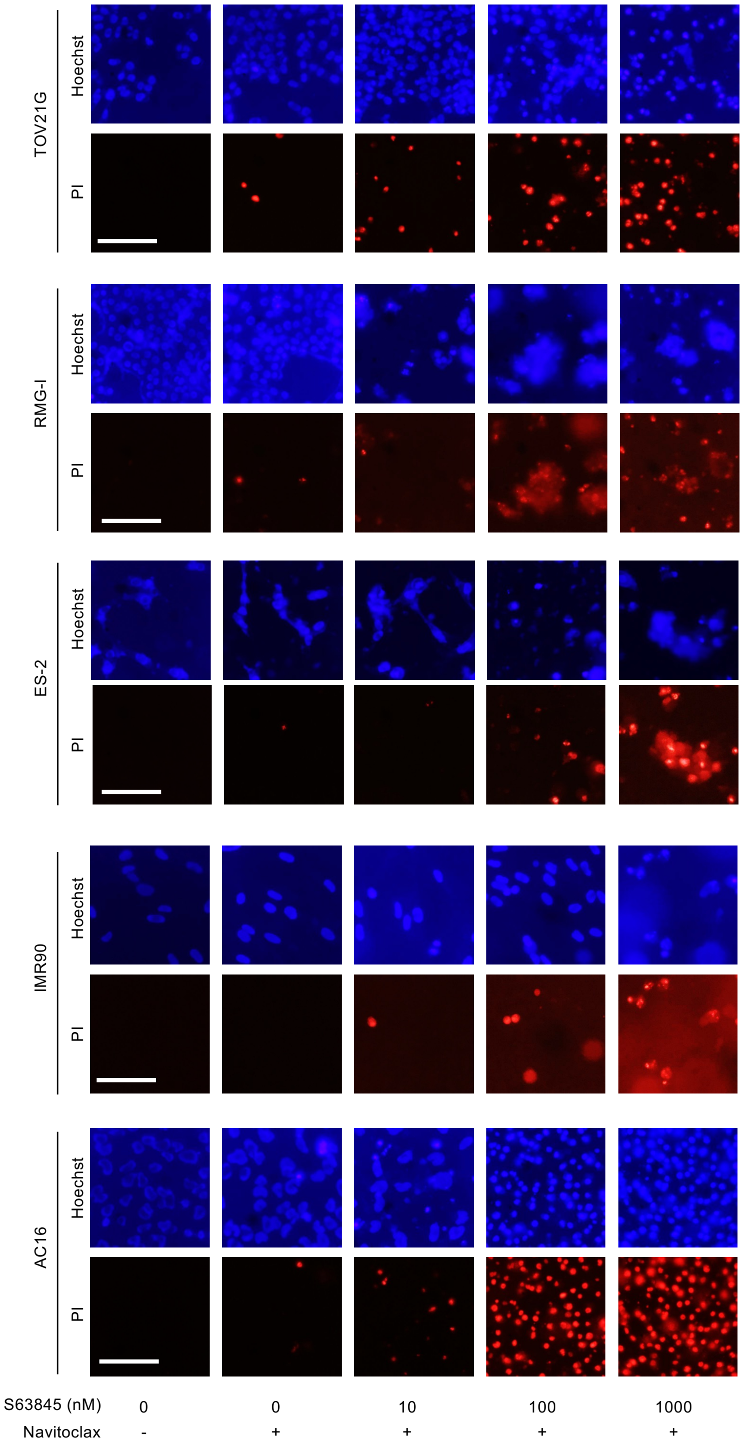

Supplementary Figure S2 (continued)

(F) Figure 5B

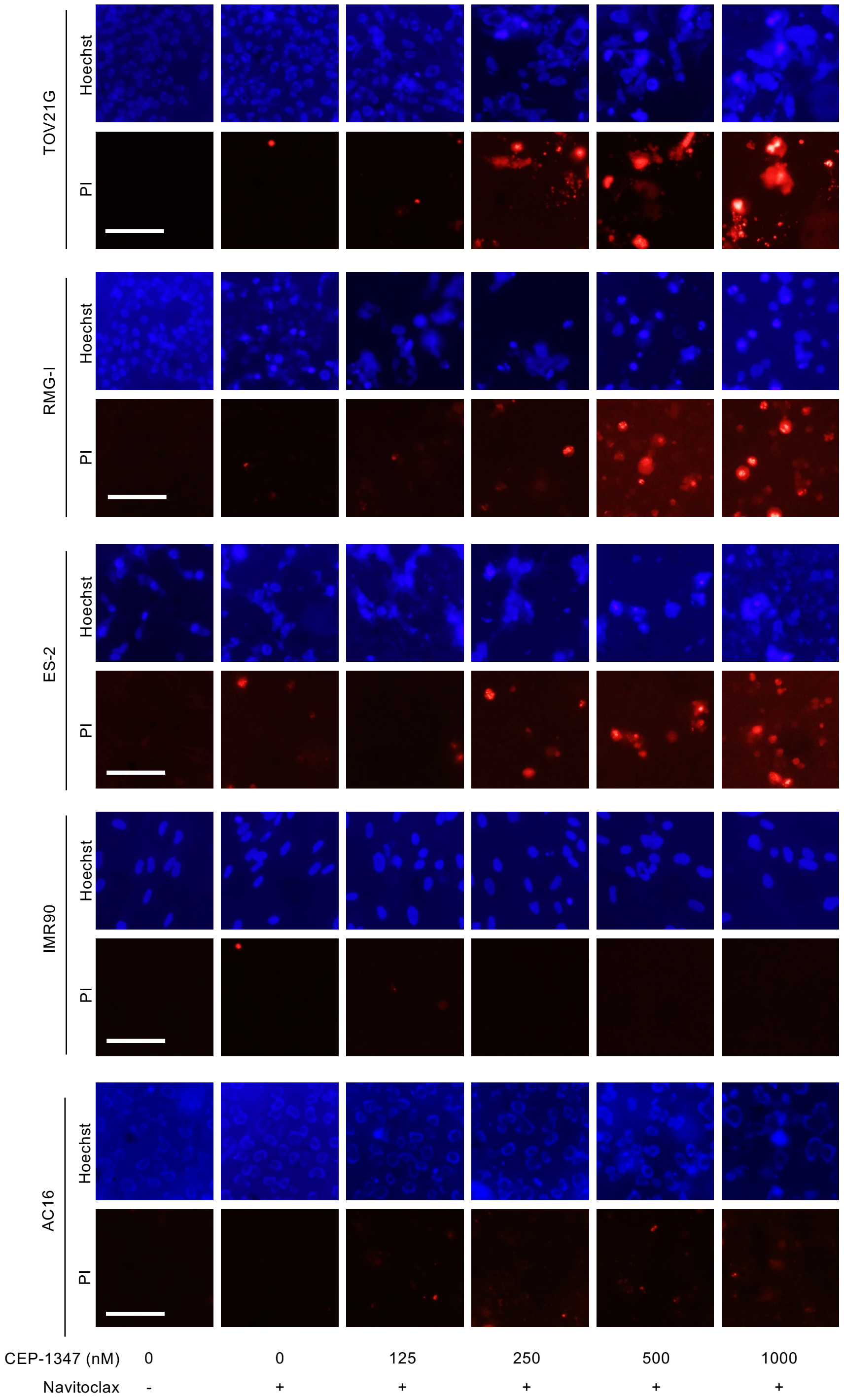

Supplementary Figure S2 (continued)

(G) Figure 5C

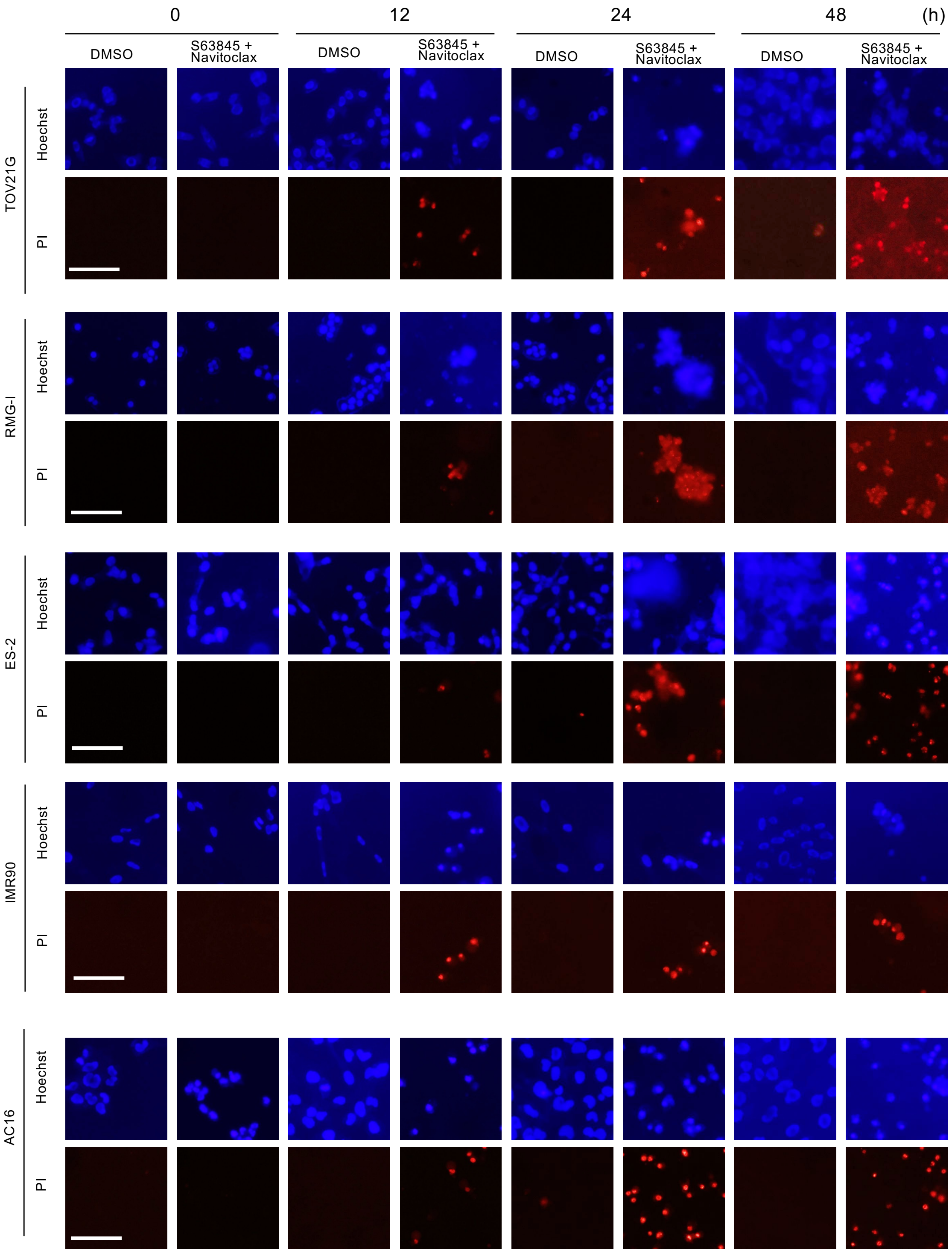

Supplementary Figure S2 (continued)

(H) Figure 5D

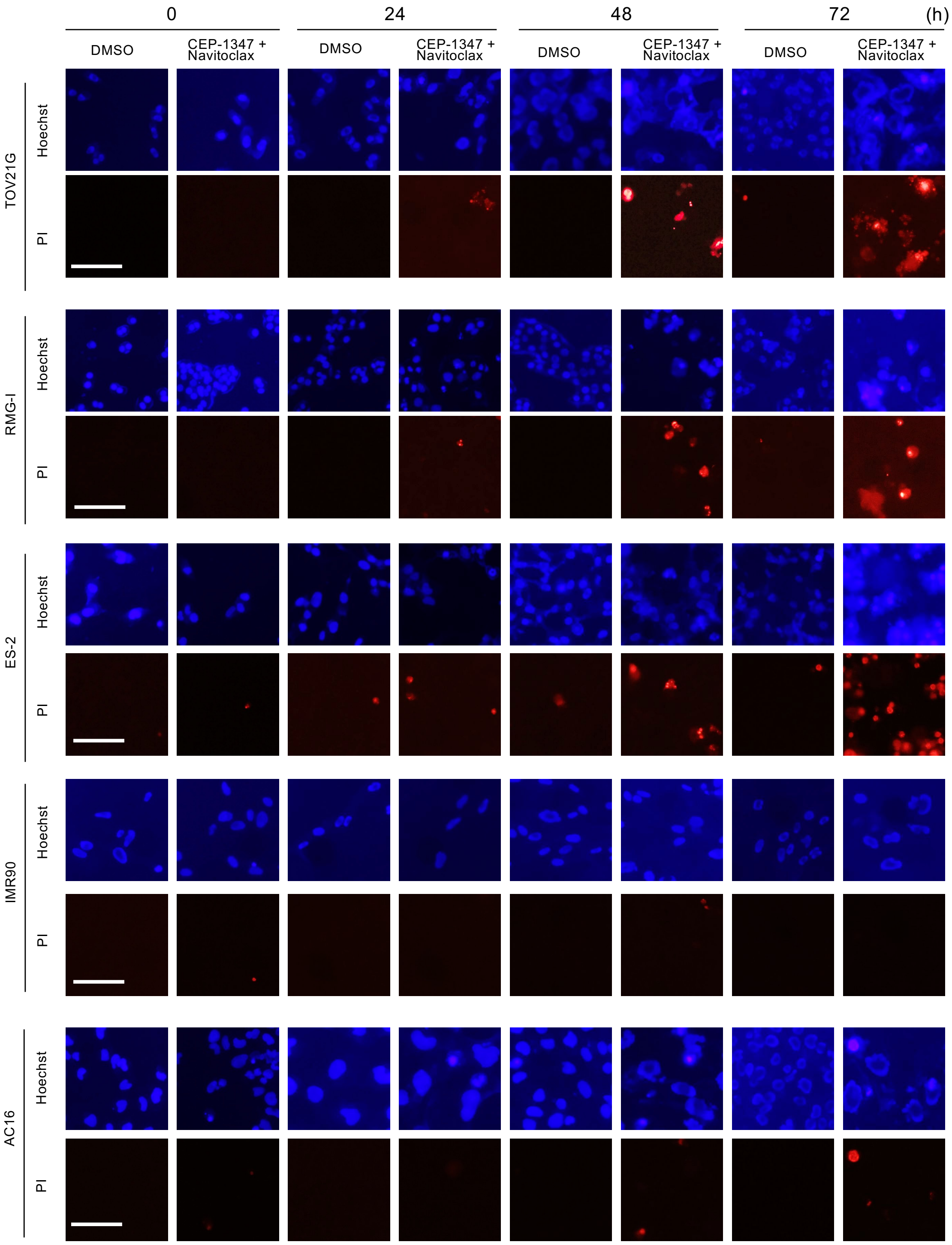

Supplementary Figure S2 (continued)

(I) Figure S1A

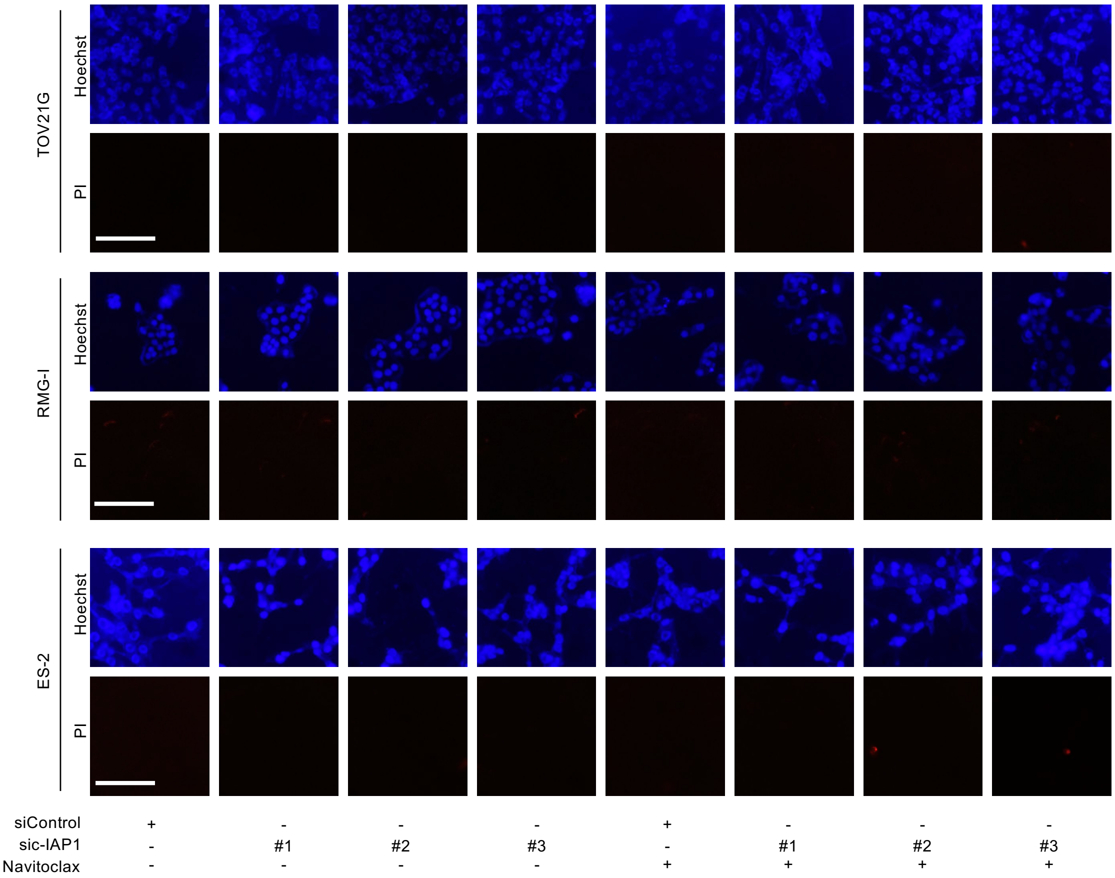

Supplementary Figure S2. Representative images of microscopic analyses. **(A)** Figure 1A. **(B)** Figure 1C. **(C)** Figure 3A. **(D)** Figure 4B. **(E)** Figure 5A. **(F)** Figure 5B. **(G)** Figure 5C. **(H)** Figure 5D. **(I)** Figure S1A. Bars; 50 µm.

Supplementary Figure S3

(A) Figure 1B

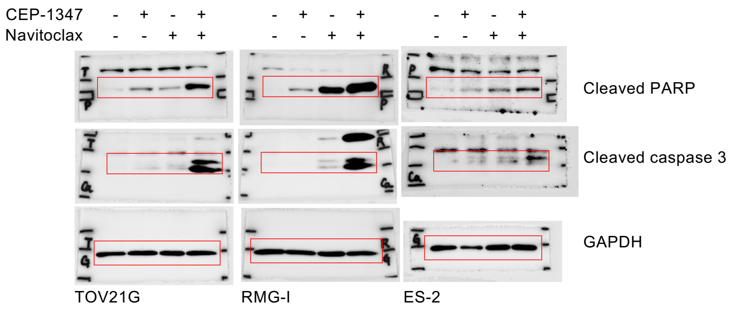

(B) Figure 1D

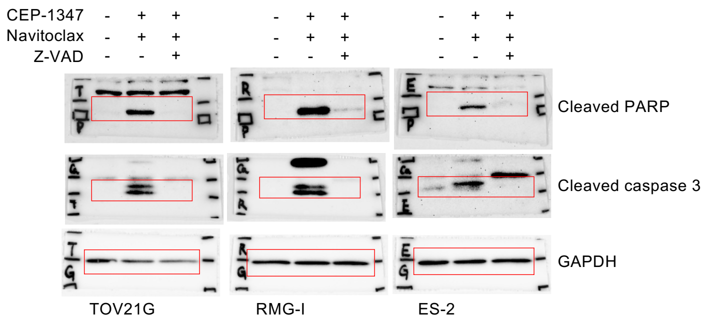

(C) Figure 2

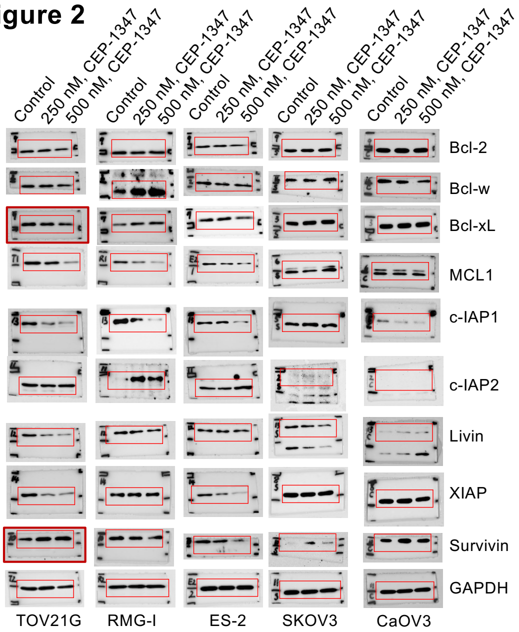

(D) Figure 3B

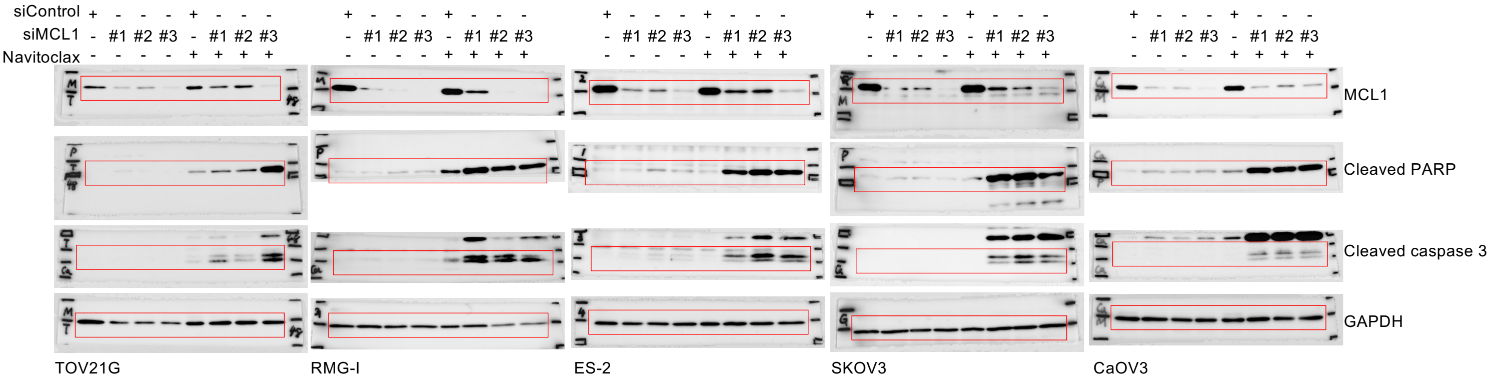

(E) Figure 4A

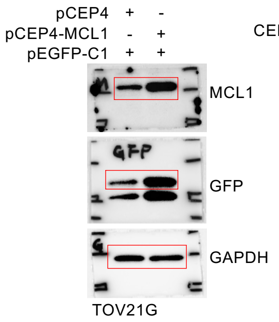

(F) Figure 5E

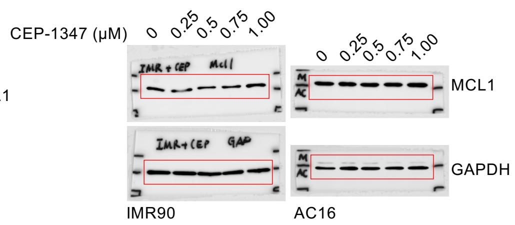

(G) Figure 5F

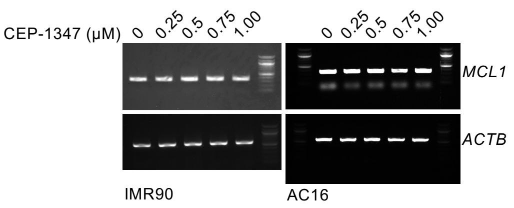

Supplementary Figure S3 (continued)

(H) Supplementary Figure S1B

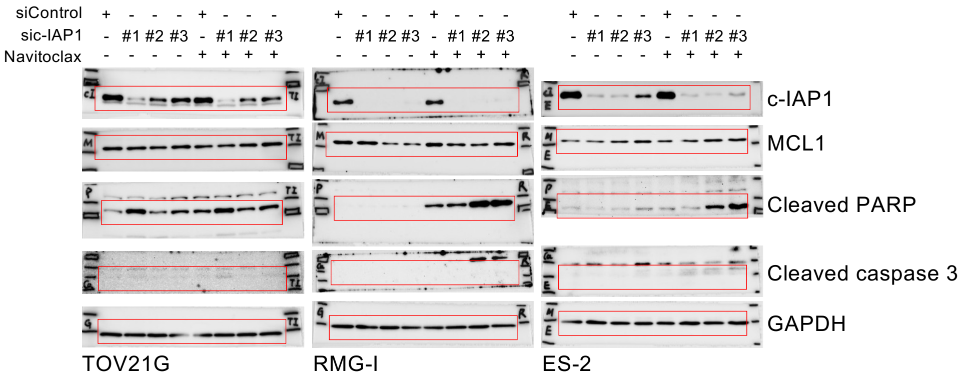

Supplementary Figure S3. Representative images of uncropped blots. **(A)** Figure 1B. **(B)** Figure 1D. **(C)** Figure 2. **(D)** Figure 3B. **(E)** Figure 4A. **(F)** Figure 5E. **(G)** Figure 5F. **(H)** Figure S1B.
